# The FOXM1/RNF26/p57 axis regulates the cell cycle to promote the aggressiveness of bladder cancer

**DOI:** 10.1038/s41419-021-04260-z

**Published:** 2021-10-14

**Authors:** Lu Yi, Haohui Wang, Wei Li, Kun Ye, Wei Xiong, Haixin Yu, Xin Jin

**Affiliations:** 1 Department of Urology, The Second Xiangya Hospital, Central South University, Changsha, Hunan 410011 China; 2 Uro-Oncology Institute of Central South University, Changsha, Hunan 410011 China; 3 Hunan Engineering Research Center of Smart and Precise Medicine, Changsha, Hunan 410011 China; 4 Cancer Center, Union Hospital, Tongji Medical College, Huazhong University of Science and Technology, Wuhan, 430022 China

**Keywords:** Oncogenes, Bladder cancer

## Abstract

Bladder cancer is one of the most lethal cancers in the world. Despite the continuous development of medical technologies and therapeutic strategies, the overall survival rate of bladder cancer has not changed significantly. Targeted therapy is a new promising method for bladder cancer treatment. Thus, an in-depth study of the molecular mechanism of the occurrence and development of bladder cancer is urgently needed to identify novel therapeutic candidates for bladder cancer. Here, bioinformatics analysis demonstrated that RNF26 was one of the risk factors for bladder cancer. Then, we showed that RNF26 is abnormally upregulated in bladder cancer cells and tissues and that higher RNF26 expression is an unfavorable prognostic factor for bladder cancer. Moreover, we found that RNF26 promotes bladder cancer progression. In addition, we showed that RNF26 expression is promoted by FOXM1 at the transcriptional level through MuvB complex. The upregulated RNF26 in turn degrades p57 (CDKN1C) to regulate the cell cycle process. Collectively, we uncovered a novel FOXM1/RNF26/p57 axis that modulates the cell cycle process and enhances the progression of bladder cancer. Thus, the FOXM1/RNF26/p57 signaling axis could be a candidate target for the treatment of bladder cancer.

## Introduction

Bladder cancer, including the muscle-invasive and non-muscle-invasive types, is mostly derived from the transitional epithelium of the urinary tract [1]. In recent decades, the incidence and mortality of bladder cancer have increased sharply. Specifically, there are ~549,000 new cases and 200,000 deaths each year worldwide [2]. Despite the continuous development of medical technologies and therapeutic strategies, the overall survival (OS) rate of bladder cancer has not changed significantly [3]. Targeted therapy is a new potential and promising method of bladder cancer treatment [4]. Therefore, in-depth study of the molecular mechanism of the occurrence and development of bladder cancer is essential for innovative treatment and ultimately for improving the prognosis and quality of life of patients.

Protein ubiquitination is an important posttranslational modification that regulates nearly all aspects of eukaryotic biology [5]. Aberrant ubiquitin signaling plays crucial roles in the initiation and development of cancer and other diseases [6]. E3 ligases are critical for this type of posttranslational modification, as they specifically bind with its substrate and catalyze the transfer of ubiquitin from an E2 enzyme to form a covalent bond with a substrate lysine [7]. RING domain E3 ubiquitin ligases are the most common E3 ligases, and their E3 activities are mainly determined by the E2-binding RING domain, which enables the formation of the E2–E3 complex [8]. The E3 ubiquitin-protein ligase RNF26, which contains a RING domain, is a transmembrane protein located in the endoplasmic reticulum [9]. RNF26 recruits and ubiquitinates scaffold p62/sequestosome 1 to retain vesicles in the perinuclear space [10]. RNF26 is upregulated ubiquitously in several human cancer cell lines, such as HL-60, HeLa S3, and MKN7, and gastric cancer tissues compared to normal controls [11]. However, the biological effect of RNF26 in cancer, especially in bladder cancer, is still elusive.

In this study, we applied bioinformatics analysis to identify RNF26 as a risk factor for bladder cancer. Then, we demonstrated that RNF26 overexpression was not only an unfavorable prognostic factor but also promoted cell growth and invasion in bladder cancer. Finally, we identified a novel FOXM1/RNF26/p57 signaling axis that modulates the cell cycle process and enhances the progression of bladder cancer.

## Materials and methods

### Data mining and bioinformatics analysis

The Cancer Genome Atlas (TCGA), ChIP-Atlas, and UbiBrowser were used for data mining and bioinformatics analysis (see Supplementary Methods for details).

### Cell lines

Bladder cancer cell lines 5637 and T24 were purchased from Yuchi Biology (Shanghai, China). Both cell lines were authenticated by short tandem repeat profiling. Cells were cultured in Dulbecco’s modified Eagle’s medium (Gibco, USA) or RPMI-1640 medium (Gibco, USA) with 10% fetal bovine serum (AC03L055, Shanghai Life-iLab Biotech, China) and kept in a 37 °C incubator supplied with 5% CO_2_.

### Short hairpin RNA (shRNA) infection and reagents

shRNAs were obtained from GeneCopoeia (HSH111798-LVRU6GP, USA), and the sequences of the shRNAs are provided in Table S1. Cells were transfected with the indicated plasmids or shRNAs using Lipofectamine 2000 (Thermo Fisher Scientific, China) according to the manufacturer’s instructions. To construct stable shRNA-infected cells, puromycin (#HY-B1743A, MedChemExpress) was used to select positive cells. The 26S proteasome inhibitor MG132 (#S2619), cycloheximide (CHX) (#S7418), and FOXM1 inhibitor (FDI-6) (#S9689) were obtained from Selleckchem.

### Co-immunoprecipitation (IP) and western blotting

For co-IP, cells harvested from 10-cm plates were lysed in RIPA buffer (#P0013C, Beyotime, China) on ice for 30 min. The supernatant was collected after centrifugation (12,000 × *g*, 15 min) and cocultured with protein A + G beads (#P2029, Beyotime, China) and IgG antibody or primary antibody at 4 °C overnight. The next day, the beads were washed with RIPA buffer six times. The protocols for the use of human tissue (12 pairs of matched bladder cancer/adjacent noncancerous tissues) were approved by the local ethics committee (the Second Xiangya Hospital, Central South University). For western blotting, the cells were harvested and lysed in RIPA buffer on ice for 30 min. The supernatant was collected after centrifugation (12,000 × *g*, 15 min). Then, 4× loading buffer was added to the supernatant and boiled in hot water (100 °C) for 10 min. The supernatant was then subjected to electrophoresis on SDS-PAGE gels. The detailed protocol was reported previously [12]. The primary antibodies used were as follows: GAPDH (Proteintech, #60004-1-Ig, 1:5000 dilution), RNF26 (Proteintech, #16802-1-AP, 1:800 dilution), p57 (Proteintech, #23317-1-AP, 1:800 dilution), p53 (Proteintech, #10442-1-AP, 1:3000 dilution), and FOXM1 (Proteintech, #13147-1-AP, 1:1000 dilution). ImageJ software (National Institutes of Health) was used to evaluate the protein levels.

### Xenografts assay

All animal experiments were approved by the ethics committee of the Second Xiangya Hospital, Central South University. BALB/c nude mice (6 weeks old) were purchased from Vital River (Beijing, China). Power analysis was used to calculate the sample size required for animal experiments and animals were randomized to the different groups. Cells infected with shControl, shRNF26, Tsin-RNF26, or shp57 (48 h after transfection) were subcutaneously injected into the left side of the backs of the mice (1 × 10^7^ cells per mouse) (*n* = 5 mice per group). A Vernier caliper was used to measure the length and width of the tumors every 2 days, and tumor volume was calculated using the formula (L × W^2^)/2. Once the mice were euthanized, the tumors were excised and weighed.

### Statistical analysis

The experimental data are presented as the mean ± standard deviation (mean ± SD). The sample size (*n*) for each statistical analysis is provided in the figure legends. GraphPad Prism 5 software was used to calculate the *P* value. Differences were considered statistically significant when the *P* values were less than 0.05.

Other methods are provided in the Supplementary Information, the primer sequences for RT-PCR and ChIP-qPCR are provided in Tables S2 and S3.

## Results

### RNF26 upregulation predicts an unfavorable prognosis in bladder cancer patients

**Fig. 1:**
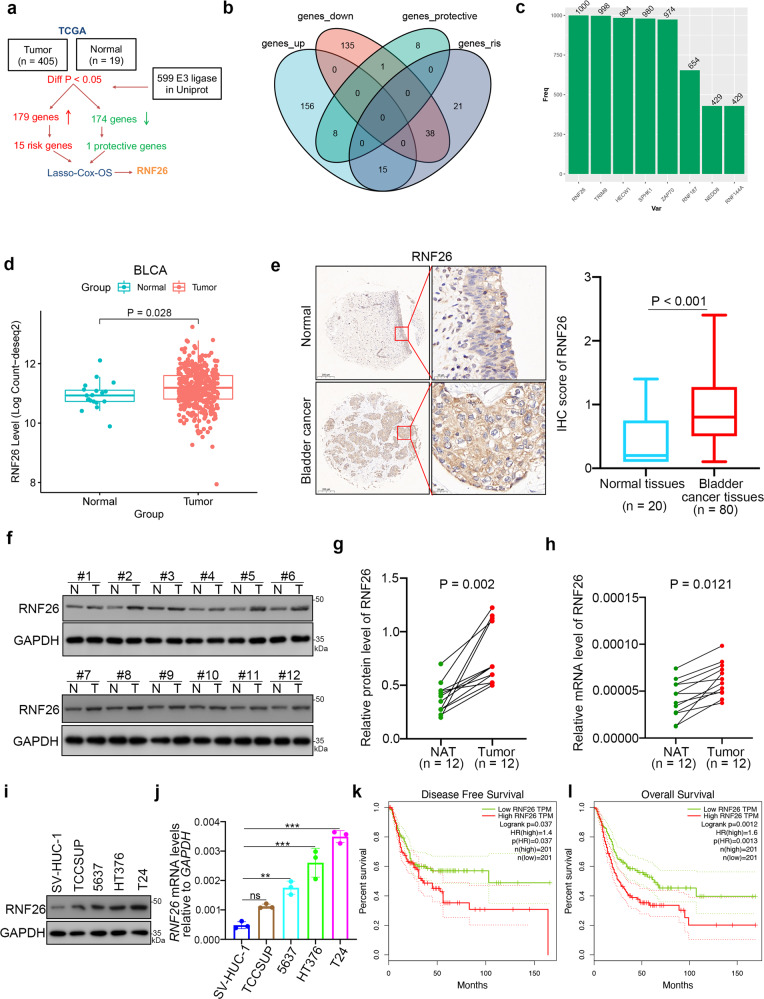
RNF26 upregulation predicts an unfavorable prognosis in bladder cancer patients. **a** Identification of RNF26 from 599 E3 ubiquitin ligases in predicting prognosis of patients with BLCA. **b** Venn diagrams showing numbers of deferentially expressed genes and prognostic genes among the 599 E3 ubiquitin ligases in TCGA-BLCA dataset. **c** Tenfold cross-validation with 1000 replications for variable selection in the LASSO-COX-OS model by minimum criteria (the 1-SE criteria). **d** Differential expression analyses of RNF26 between tumor and normal tissues in TCGA-BLCA dataset. **e** IHC analysis of the tissue microarray by staining the RNF26 antibody. The typical image and expression level of RNF26 in the nontumor tissue and bladder cancer tissue were shown. *P* values as indicated. The protein expression levels (**f** and **g**) and mRNA levels (**h**) of RNF26 in the adjacent nontumor bladder tissues (*n* = 12) and bladder cancer tissues (*n* = 12) were analyzed by the western blot (**f**) and RT-qPCR assay (**h**). The protein levels of RNF26 were quantified by the imageJ software. *P* values as indicated in (**g**) and (**h**). **i**, **j** Western blot and RT-qPCR were applied to examine the protein and mRNA expression levels of RNF26 in the urinary tract normal cell lines and bladder cancer cell lines. Data present as mean ± SD with three replicates. Ns not significant; **P < 0.01; ***P < 0.001. Kaplan–Meier analysis with two-sided log-rank test was conducted using GEPIA2 to evaluate the differences in RFS (**k**) and OS (**l**) between the patients with high and low expression of RNF26 in TCGA-BLCA dataset.

Through screening UniProt data, 599 E3 ubiquitin ligases were obtained (Fig. 1a). Among the 599 E3 ligases, 179 were upregulated and 174 were downregulated between cancer versus normal samples in the TCGA-BLCA dataset (*P* < 0.05). Using OS as the outcome, univariate Cox regression analysis in the TCGA-BLCA dataset showed that 15 of the 179 genes were risk-related genes (HR > 1, *P* < 0.05) and 1 of the 174 genes was a protective gene (HR < 1, *P* < 0.05) (Fig. 1a, b). Least absolute shrinkage and selection operator (LASSO)-Cox regression analysis with 1000 replications for the 16 prognostic genes in the TCGA-BLCA dataset further showed that RNF26 may be a key gene related to the OS of bladder cancer (Fig. 1c). Furthermore, we also showed that RNF26 expression was upregulated in the bladder cancer tissues compared to nontumor bladder tissues (Fig. 1d). Then, we performed IHC staining to detect the protein level of RNF26 in bladder cancer tissues and nontumor bladder tissues in a tissue microarray for a cohort of patients with bladder cancer. Representative images are shown in Fig. 1e. Similar to the above findings, RNF26 had a higher expression level in bladder cancer tissues than in nontumor bladder tissues (Fig. 1e). Moreover, we analyzed the protein and mRNA levels of RNF26 in samples derived from patients with bladder cancer. We also found that RNF26 was upregulated in bladder cancer tissues compared with adjacent tissues (Fig. 1e–g). Consistently, we showed that the expression levels of RNF26 were higher in bladder cancer cell lines than in immortalized human bladder epithelial cells (SV-HUC-1) (Fig. 1h, i). Then, the Gene Expression Profiling Interactive Analysis (GEPIA) web tool indicated that high expression of RNF26 was associated with shorter disease-free survival and OS times in bladder cancer (Fig. 1j, k). Together, our results suggest that RNF26 is abnormally overexpressed in bladder cancer and correlated with a poor prognosis in bladder cancer patients.

### RNF26 promotes the proliferation and invasion of bladder cancer cells

**Fig. 2:**
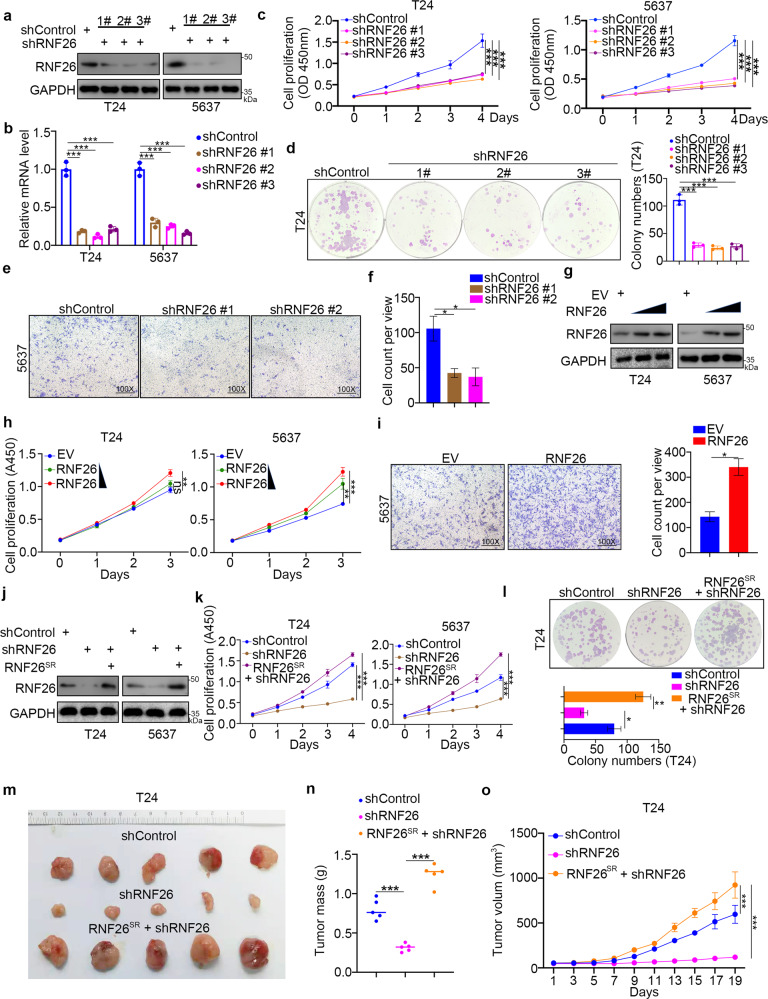
RNF26 promotes the proliferation and invasion of bladder cancer cells. **a–f** T24 and 5637 cells were infected with shControl or shRNF26 for 72 h. After puromycin selection, cells were harvested for western blot analysis (**a**), RT-qPCR analysis (**b**), CCK-8 assay (**c**), colony formation assay (**d**), and transwell assay (**e**). Data present as mean ± SD with three replicates. *P < 0.05; ***P < 0.001. **g–i** T24 and 5637 cells were transfected with indicated plasmids. Forty-eight hours post transfection, cells were collected for western blot analysis (**g**), CCK-8 assay (**h**), and transwell assay (**i**). Data present as mean ± SD with three replicates. Ns not significant; *P < 0.05; **P < 0.01; ***P < 0.001. **j–o** T24 and 5637 cells were infected with indicated constructs for 72 h. After puromycin selection, cells were harvested for the western blot analysis (**j**), CCK-8 assay (**k**), colony formation assay (**l**), and subcutaneous xenografts assay (**m**–**o**). For **k** and **l**, data present as mean ± SD with three replicates. *P < 0.05; **P < 0.01; ***P < 0.001. For **m**–**o**, data present as mean ± SD with five replicates. ***P < 0.001.

Since the clinicopathological features related to RNF26 expression indicated RNF26 as an oncogenic protein in bladder cancer, we investigated the biological effect of RNF26 in bladder cancer cells. Three different shRNAs were applied to knockdown RNF26 in T24 and 5637 cells (Fig. 2a, b). The CCK-8 assay, colony formation assay, and Transwell assay indicated that RNF26 silencing blocked bladder cancer cell growth and invasion (Fig. 2c–f). In contrast, overexpression of RNF26 enhanced the growth and invasion ability of bladder cancer cells (Fig. 2g–i). Moreover, we also showed that recurring the expression of RNF26 after knockdown of RNF26 by infection with an shRNA-resistant Tsin-RNF26 construct reversed the growth-decreasing effect induced by RNF26 knockdown (Fig. 2j–l). Furthermore, xenograft assays showed that knockdown of RNF26 inhibited bladder cancer tumor growth. However, rescue of RNF26 expression resulted in increased tumor growth (Fig. 2m–o). Therefore, our data indicate that RNF26 is responsible for the progression of bladder cancer in vitro and in vivo.

### FOXM1 contributes to RNF26 overexpression in bladder cancer cells

**Fig. 3:**
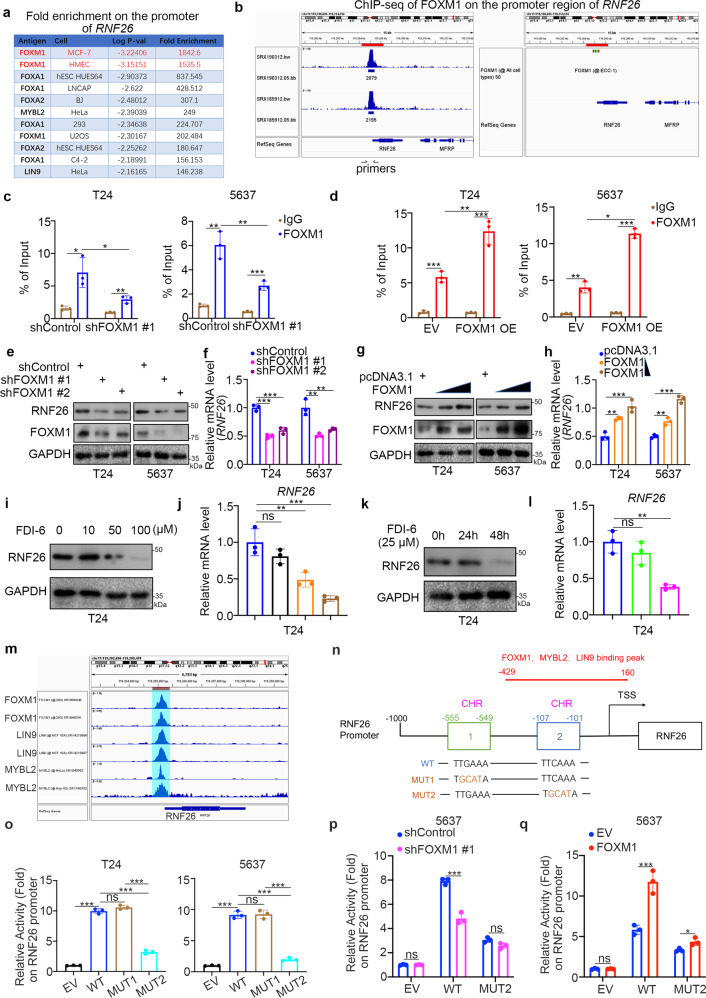
FOXM1 contributes to RNF26 overexpression in bladder cancer cells. **a** Enrichment analysis by ChIP-Atlas predicted the top five transcription factors bound to the promoter region of *RNF26* within ±1000 bp from TSS. **b** The ChIP-seq of FOXM1 on the promoter region of *RNF26*. **c** T24 and 5637 cells were infected with indicated shRNAs for 72 h. The ChIP-qPCR were performed by using the IgG and FOXM1 antibodies in the T24 and 5637 cells. Data present as mean ± SD with three replicates. *P < 0.05; **P < 0.01; ***P < 0.001. **d** T24 and 5637 cells were transfected with indicated plasmids for 24 h. The ChIP-qPCR were performed by using the IgG and FOXM1 antibodies in the T24 and 5637 cells. Data present as mean ± SD with three replicates. *P < 0.05; **P < 0.01; ***P < 0.001. **e**, **f** T24 and 5637 cells were infected with indicated shRNAs for 72 h. Cells were harvested for the western blot analysis (**e**) and RT-qPCR analysis (**f**). Data present as mean ± SD with three replicates. **P < 0.01; ***P < 0.001. **g**, **h** T24 and 5637 cells were transfected with indicated plasmids for 24 h. Cells were harvested for the western blot analysis (**g**) and RT-qPCR analysis (**h**). Data present as mean ± SD with three replicates. **P < 0.01; ***P < 0.001. **i**, **j** T24 cells were treated with 0, 10, 50, 100 μM FDI-6 for 24 h. Cells were harvested for the western blot analysis (**i**) and RT-qPCR analysis (**j**). Data present as mean ± SD with three replicates. Ns not significant; **P < 0.01; ***P < 0.001. **k**, **l** T24 cells were treated with 25 μM FDI-6 for 0, 24, and 48 h. Cells were harvested for the western blot analysis (**k**) and RT-qPCR analysis (**l**). Data present as mean ± SD with three replicates. Ns, not significant; **P < 0.01. **m** the ChIP-seq of FOXM1, LIN9, and MYBL2 on the promoter region of *RNF26*. **n** diagram demonstrated the sequence and position of CHR element, the FOXM1, LIN9, and MYBL2 binding peak, in the RNF26 promoter. TSS transcriptional start site, WT wild type, MUT mutant type. **o** T24 and 5637 cells were transfected with empty vector, GV592-RNF26 plasmids WT, MUT1, or MUT2 for 48 h. Cells were harvested and the activity of RNF26 promoter was measured. Data present as mean ± SD with three replicates. Ns not significant; ***P < 0.001. **p** 5637 cells were infected with shControl or shFOXM1 #1 for 48 h. Then, cells were transfected with EV, GV592-RNF26 plasmids WT, or MUT2 for another 48 h. Cells were harvested and the activity of RNF26 promoter was measured. Data present as mean ± SD with three replicates. Ns not significant; ***P < 0.001. **q** 5637 cell were transfected with EV or FOXM1 plasmids for 24 h. Then, cells were transfected with EV, GV592-RNF26 plasmids WT, or MUT2 for another 48 h. Cells were harvested and the activity of RNF26 promoter was measured. Data present as mean ± SD with three replicates. Ns not significant; *P < 0.05; ***P < 0.001.

Since we identified that RNF26 was aberrantly overexpressed in bladder cancer cell lines and patient tissues (Fig. 1), we sought to explore the underlying regulatory mechanism of RNF26. We performed bioinformatic analysis to identify the potential transcription factors of RNF26. Using existing ChIP-seq datasets in ChIP-Atlas, we found that there were a number of transcription factors binding to the promoter region of *RNF26* (Fig. 3a). It is worth noting that FOXM1 has a binding peak in the promoter region of *RNF26*, and this peak showed the highest fold enrichment and lowest *P* values (Fig. 3a, b). Moreover, it has been documented that activated FOXM1 is critical for the progression and drug resistance of bladder cancer [13, 14]. Thus, we were curious about whether FOXM1 is responsible for the transcriptional regulation of RNF26 in bladder cancer. Then, we performed ChIP-qPCR by using FOXM1 or IgG antibodies and found that FOXM1 bound to the promoter region of *RNF26* in T24 and 5637 cells (Fig. 3c, d). Moreover, we demonstrated that knockdown of FOXM1 decreased its binding with the promoter of RNF26 and reduced the expression of RNF26 in both T24 and 5637 cells (Fig. 3e, f). In contrast, ectopic overexpression of FOXM1 enhanced FOXM1 binding to the promoter of RNF26 and increased the RNF26 expression level in bladder cancer cells (Fig. 3g, h). Furthermore, we treated bladder cancer cells with a FOXM1-specific inhibitor (FDI-6) to detect changes in RNF26 in T24 cells. It was not surprising that FDI-6 decreased the protein and mRNA levels of RNF26 in a dose- and time-dependent manner (Fig. 3i–l). In addition, the correlation between FOXM1 and RNF26 in the cancer patient samples was analyzed through the GEPIA web tool. Consistently, we found that FOXM1 was positively correlated with RNF26 in multiple types of malignant tumors, including bladder cancer, colon cancer, cervical cancer, breast cancer, prostate cancer, pancreatic cancer, liver cancer, and gastric cancer (Supplementary Fig. 1).

However, we analyzed the promoter region of RNF26 and found that there was no FOXM1 consensus binding motif (TAACA) [15] in the promoter region of RNF26. It has been reported that FOXM1 can interact with the MuvB complex to indirectly regulated the expression of downstream target genes expression, including CCNB1 and RNF26 [16, 17]. Furthermore, the ChIP-Atlas analysis indicated that LIN9, the major component of MuvB complex [18], also bound to the promoter region of RNF26 (Fig. 3a). In addition, MYBL2, which, like FOXM1, interacts with MuvB and activates the transcription of target promoters indirectly [17, 18], also bound to the promoter region of RNF26 (Fig. 3a). Notably, we showed that the binding regions for FOXM1, LIN9, and MYBL2 in the RNF26 promoter was overlapped (Fig. 3m). Since FOXM1 interacted with MuvB/LIN54 complexes to directly bind to the cell cycle genes homology region (CHR) element, we found that there were two CHR motifs (TTYRAA) [19] in the promoter region of RNF26 (Fig. 3n). We cloned DNA sequences containing these two CHR motifs (wild-type (WT) and CHR mutants (MUT1 and MUT2)) to generated three GV592-RNF26 promoter plasmids as indicated (Fig. 3n). We transfected T24 and 5637 cells with empty vector or the GV592-RNF26 plasmids and showed that the luciferase activity of the WT GV592-RNF26-promoter was increased (Fig. 3o). Furthermore, the luciferase activity of the MUT2 GV592-RNF26-promoter was lower than that of the WT and MUT1 GV592-RNF26-promoter, but there was no difference between the luciferase activity of the MUT1 GV592-RNF26 promoter and the WT GV592-RNF26 promoter in T24 and 5637 cells (Fig. 3o). Moreover, knockdown of FOXM1 markedly decreased the luciferase activity of the WT GV592-RNF26 promoter but had no effect on the activity of the MUT2 GV592-RNF26 promoter in 5637 cells (Fig. 3p). In contrast, overexpression of FOXM1 significantly increased the luciferase activity of the WT GV592-RNF26 promoter and slightly increased the activity of the MUT2 GV592-RNF26 promoter in 5637 cells (Fig. 3q). Therefore, our data suggest that FOXM1 regulates the expression of RNF26 in bladder cancer.

### RNF26 regulates the cell cycle and interacts with p57 in bladder cancer cells

**Fig. 4:**
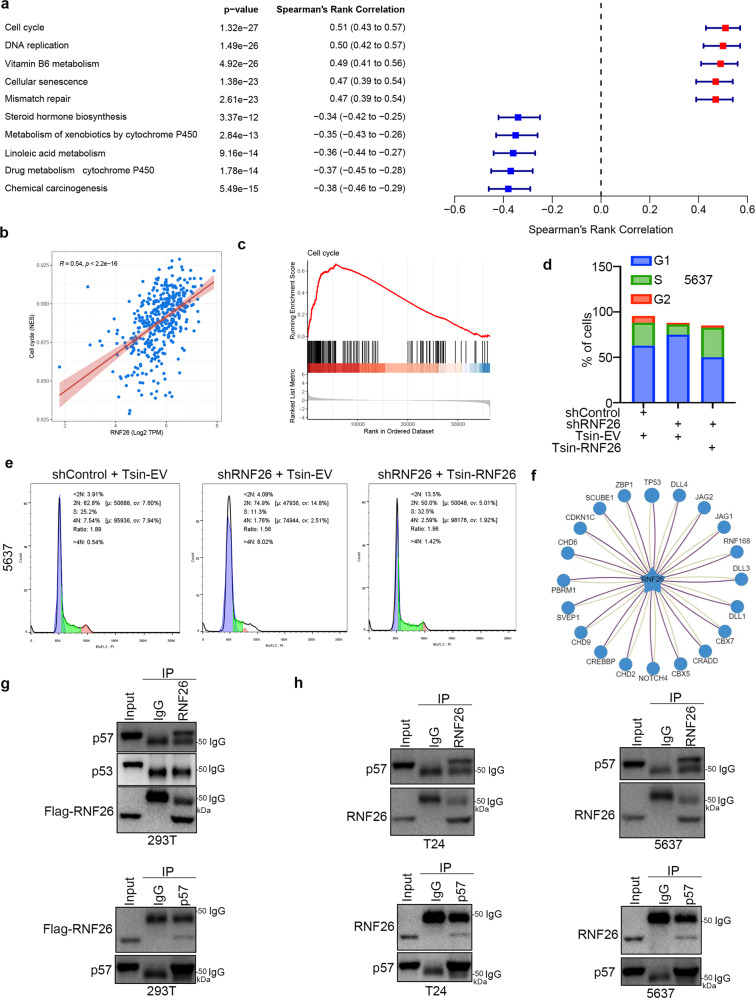
RNF26 regulates the cell cycle and interacts with p57 in bladder cancer cells. **a** Single-sample GSEA (ssGSEA) showed the top five pathways positively correlated with RNF26 and the top 5 pathways negatively correlated with RNF26 in TCGA-BLCA dataset. **b** Correlation analysis between RNF26 and cell cycle pathway in TCGA-BLCA dataset. **c** Cell cycle pathway was activated by the overexpression of RNF26 in TCGA-BLCA dataset. **d**, **e** 5637 cells were infected with indicated constructs for 72 h. After puromycin selection, cells were subjected to cell cycle analysis. **f** UbiBrowser showed the top 20 substrate proteins that might interact with RNF26. **g** 293T cells were transfected with Flag-RNF26 for 24 h. Cells were harvested and underwent co-IP assay by using the RNF26 or p57 antibodies, respectively. **h** T24 and 5637 were harvested and underwent co-IP assay by using the RNF26 or p57 antibodies respectively.

To explore the underlying mechanism of how RNF26 enhanced the progression of bladder cancer cells, we performed gene set enrichment analysis (GSEA) of the TCGA-BLCA dataset to study the pathways in which RNF26 is involved. We showed that the cell cycle signaling pathway had the most positive association with RNF26 in bladder cancer cells (Fig. 4a–c). Consistently, we performed cell cycle analysis, which demonstrated that knockdown of RNF26 blocked G1/S phase progression and that rescuing RNF26 expression by Tsin-RNF26 infection reversed this effect (Fig. 4d, e). In addition, UbiBrowser showed that RNF26 might interact with multiple types of substrate proteins (Fig. 4f). Among these proteins, p53 and CDKN1C (p57) are crucial for regulating the cell cycle process [17, 20]. Thus, we performed reciprocal IP to determine which protein truly bound to RNF26 in cancer cells. Intriguingly, we demonstrated that p57, not p53, could interact with RNF26 in bladder cancer cells (Fig. 4g, h). Taken together, our results showed that RNF26 plays an important role in modulating the cell cycle process and interacts with p57 in bladder cancer cells.

### RNF26 is the bona fide E3 ligase of p57

**Fig. 5:**
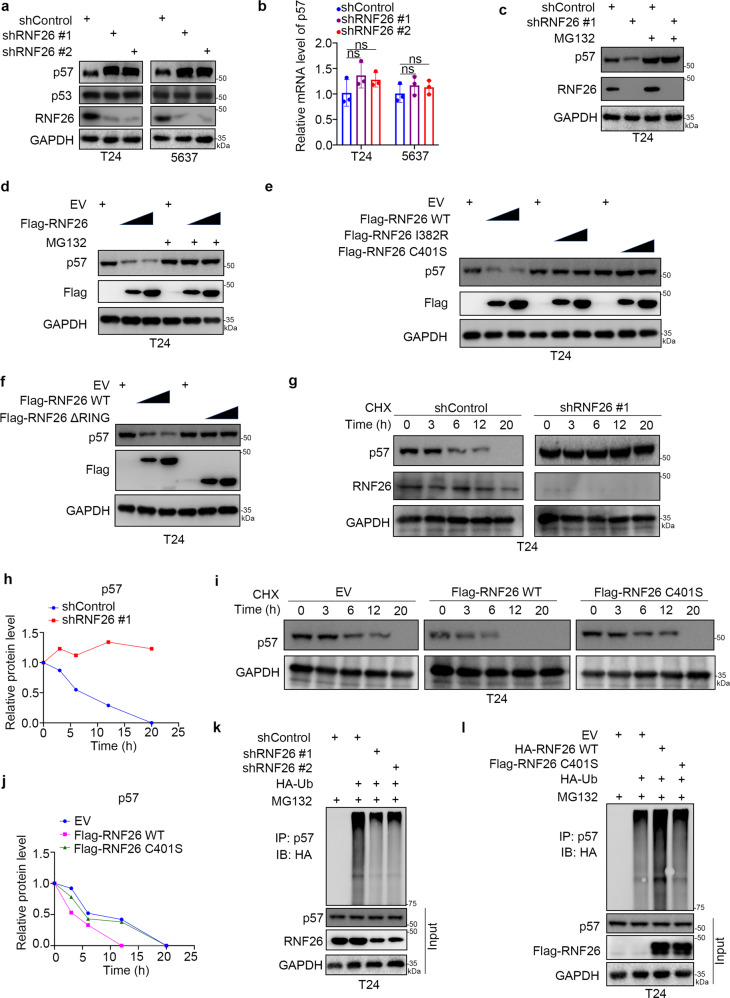
RNF26 is the bona fide E3 ligase of p57. **a**, **b** T24 and 5637 cells were infected with indicated shRNAs for 72 h. After puromycin selection, cells were harvested for western blot analysis (**a**) and RT-qPCR assay (**b**). Data present as mean ± SD with three replicates. Ns not significant. **c** T24 cells were infected with indicated shRNAs for 72 h. Cells were harvested for western blot analysis before treated with or without MG132 for 8 h. **d** T24 cells were transfected with indicated plasmids for 24 h. Cells were harvested for western blot analysis before treated with or without MG132 for 8 h. **e** T24 cells were transfected with indicated plasmids for 24 h. Cells were harvested for western blot analysis. **f** T24 cells were transfected with indicated plasmids for 24 h. Cells were harvested for western blot analysis. **g**, **h** T24 cells were infected with the indicated shRNAs. After 72 h, cells were treated with cycloheximide (CHX), and cells were collected for western blot analysis at different time points. **i**, **j** T24 cells were transfected with the indicated plasmids. After 24 h, cells were treated with CHX, and cells were collected for western blot analysis at different time points. **k** T24 cells were infected with the indicated shRNAs. After 72 h, cells were collected for western blot after treatment with MG132 for 8 h. **l** T24 cells were transfected with the indicated plasmids. After 24 h, cells were collected for western blot after treatment with MG132 for 8 h.

Studies have shown that p57 functions as a cyclin-dependent kinase (CDK) inhibitor of G1 phase cell cycle arrest [21]. Similarly, the above data indicated that RNF26 silencing inhibited the progression of the cell cycle from G1 to S phase. Since RNF26 is an E3 ligase and RNF26 interacts with p57, we hypothesized that RNF26 might degrade p57 to regulate the cell cycle process. It is worth noting that RNF26 knockdown by two different shRNAs elevated the protein levels of p57 but not p53 in both T24 and 5637 cells (Fig. 5a). Interestingly, knockdown of RNF26 had no effect on the mRNA level of RNF26 in bladder cancer cells (Fig. 5b). Moreover, we showed that 26S proteasome inhibitor (MG132) treatment attenuated the upregulation of p57 induced by RNF26 silencing (Fig. 5c). In contrast, ectopic overexpression of RNF26 reduced p57 protein levels, and this process could be blocked by MG132 in T24 cells (Fig. 5d). Furthermore, transfection with the mutants I382R and C401S and the delta RING mutant ∆RING, which have been reported to decrease or abolish the E3 ligase activity of RNF26 [10, 22], failed to downregulate the protein level of p57 in T24 cells (Fig. 5e, f). In addition, we demonstrated that knockdown of RNF26 prolonged the half-life of the p57 protein (Fig. 5g, h). However, overexpression of wild-type RNF26 but not the RNF26 C401S mutant shortened the half-life of the p57 protein in T24 cells (Fig. 5i, j). It was not surprising that knockdown of RNF26 decreased the polyubiquitination levels of p57, while overexpression of RNF26 increased the polyubiquitination levels of p57 in T24 cells (Fig. 5k, l). Together, these data suggest that RNF26 acts as an E3 ligase to degrade p57 in bladder cancer cells.

### RNF26 regulates the progression of bladder cancer cells through p57

**Fig. 6:**
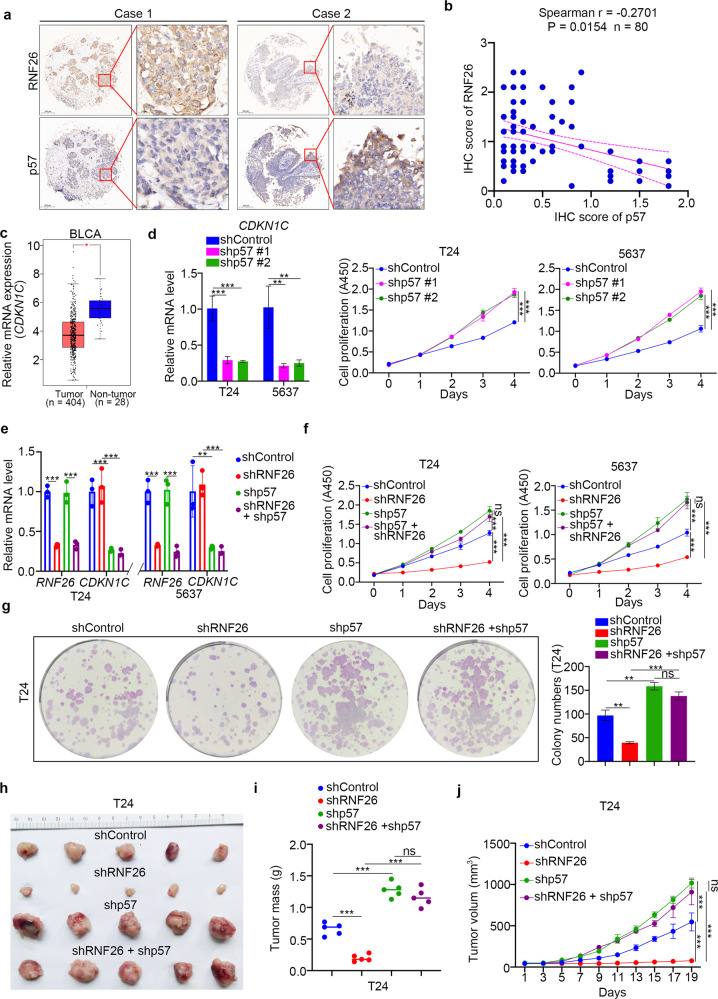
RNF26 regulates the progression of bladder cancer cells through p57. **a**, **b** Bladder cancer tissue microarray stained for RNF26 and p57. Representative images are shown in (**a**). The correlation of RNF26 and p57 levels is shown in (**b**); *P* values are also shown in the figure. **c** the GEPIA web tool was used to analyze the CDKN1C expression level in the bladder cancer. *P < 0.01. **d** T24 and 5637 cells were infected with indicated shRNAs for 72 h. Cells were collected for RT-qPCR analysis and CCK-8 assay. Data present as mean ± SD with three replicates. **P < 0.01; ***P < 0.001. **e–j** T24 and 5637 cells were infected with indicated constructs for 72 h. After puromycin selection, cells were harvested for the RT-qPCR analysis (**e**), CCK-8 assay (**f**), colony formation assay (**g**), and subcutaneous xenografts assay (**h**–**j**). For **e**, **f**, and **g**, data present as mean ± SD with three replicates. Ns not significant; **P < 0.01; ***P < 0.001. For **h**–**j**, data present as mean ± SD with five replicates. Ns not significant; ***P < 0.001.

To further study the relationship between RNF26 and p57 in bladder cancer, we performed IHC analysis by staining RNF26 and p57 in a tissue microarray derived from a cohort of patients with bladder cancer (Fig. 6a). The results showed that there was a negative correlation between RNF26 and p57 levels in the tissues of patients with bladder cancer, although the correlation is not so appreciable (Spearman *r* = −0.2701, *P* = 0.0154) (Fig. 6b). In addition, GEPIA web tool (http://gepia2.cancer-pku.cn/#index) analysis indicated that CDKN1C (p57) was downregulated in bladder cancer (Fig. 6c). Silencing p57 increased the growth of bladder cancer cells (Fig. 6d). Then, we aimed to explore whether RNF26 promotes bladder cancer cell proliferation via p57. We established bladder cancer cells with knockdown of RNF26 or p57 alone or with simultaneous knockdown of RNF26 and p57 (Fig. 6e). We showed that co-knockdown of RNF26 and p57 attenuated the tumor growth inhibition effect of RNF26 knockdown in vivo and in vitro (Fig. 6f–j). Therefore, our results indicate that p57 is the downstream effector of RNF26 for modulating the proliferation of bladder cancer cells.

## Discussion

The tumorigenesis of bladder cancer is due to alterations in multiple types of molecular pathways, which disrupt the homeostasis of cancer cells [23]. Among them, there are two major signaling pathways: the dysregulation of Ras-mitogen-activated protein kinase signaling is responsible for the low grade of bladder cancer, and alteration of the cell cycle pathway is common in the invasive stage of bladder cancer [24]. The dysregulation of key proteins associated with cell cycle modulation in bladder cancer mainly involves the p53 pathway and CDK/retinoblastoma signaling pathway [24, 25]. In this study, bioinformatics analysis indicated that RNF26 might be closely associated with p53 and CDKN1C. Further experiments suggested that CDKN1C might interact with RNF26 in bladder cancer cells. CDKN1C (p57) belongs to the Cip/Kip family and functions as a CDK inhibitor. P57 plays a vital role in regulating cell differentiation during neuronal development and erythropoiesis by controlling the cell cycle process [26]. In addition, p57 is reported to modulate cytoskeletal organization, cell migration, and genome expression [26]. p57 is critically involved in several hallmarks of cancer, including apoptosis, cell invasion and metastasis, tumor differentiation, and angiogenesis [27]. Moreover, p57 is ubiquitously downregulated in cancers due to epigenetic repression [28]. It has been reported that DNA methyltransferase 1 (DNMT1) and DNMT3 catalyze the promoter methylation of *CDKN1C* [29]. Moreover, histone hypoacetylation (mainly H3K9/K14) in the promoter of *CDKN1C* induced by the overexpression of HDAC1 and HDAC2 in cancer cells is another reason for the low expression of CDKN1C in cancers [30, 31]. Furthermore, the increase in H3K27me3 due to polycomb repressive complex 2 suppresses CDKN1C expression in Schwann cells [32]. To date, the study of the regulation of CDKN1C has been mostly focused on the transcriptional level. Here, we uncovered the regulatory mechanism of p57 at the posttranscriptional level. We showed that RNF26 could bind with and degrade p57 in bladder cancer cells. In addition, we believe that further studies will reveal the regulatory mechanism involved in the stability of the p57 protein.

Deregulation of E3 ubiquitin ligases is the hallmark of bladder cancer initiation, development, and progression [33]. Numerous E3 ubiquitin ligases, such as cIAP2 [34], FBW7 [35], RNF126 [36], and HUWE1 [37], have been documented to be associated with breast cancer tumorigenesis and drug resistance. In this study, we used LASSO regression analysis to identify that RNF26 might be an E3 ligase that is crucial for the progression of bladder cancer. RNF26 belongs to the RING domain family of proteins, which plays a key role in organizing the endosomal pathway for efficient cargo transport by mediating the ubiquitination of SQSTM1 [10]. Moreover, RNF26 was reported to limit the type I interferon response by promoting the autophagic degradation of IRF3 [22]. In addition, RNF26 could also stabilize TMEM173/STING, which catalyzes the formation of K11-linked polyubiquitin chains on TMEM173/STING at lysine 150 to prevent its degradation by RNF5 [22]. The cancer-related role of RNF26 in bladder cancer has never been mentioned. Here, we demonstrated that RNF26 enhances the proliferation and invasion of bladder cancer cells. We further showed that RNF26 degrades p57 to promote cell cycle transition and bladder cancer cell proliferation. Besides, RNF26 expression has been reported to upregulated ubiquitously in several human cancer cell lines, including human promyelocytic leukemia (HL-60) cells, human cervical cancer (HeLa S3) cells, and human gastric cancer (MKN7) cells, and gastric cancer tissues, which implies that RNF26 might have the same cancer-related function in other tissues and cancers [11]. In addition, our results indicate that RNF26 might have a close relationship with other proteins (Fig. 4). Therefore, the specific role of RNF26 in cancers, especially bladder cancer, needs to be further studied.

**Fig. 7:**
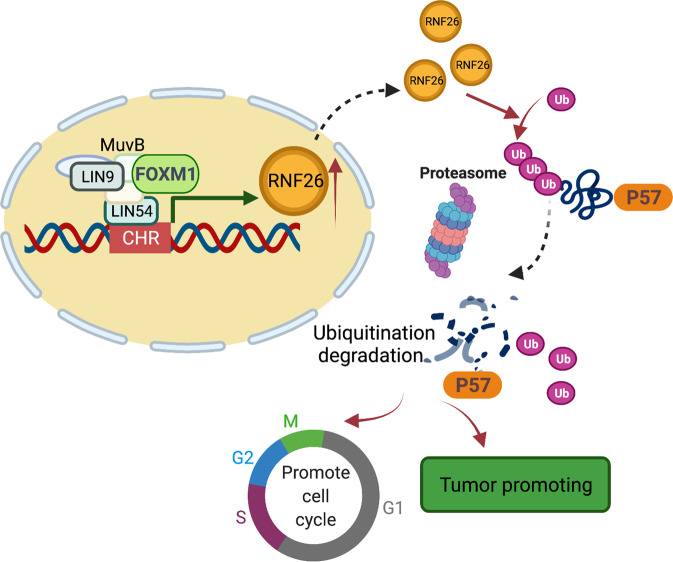
A hypothesis model depicted that the abnormal expressed FOXM1 bound to the promoter of RNF26 and initiated the transcription of RNF26 through MuvB complex in the bladder cancer cells. The upregulated RNF26 interacted with p57 to degrade p57 and enhance the progression of bladder cancer cells.

Collectively, we employed bioinformatics analysis, which showed that RNF26 is abnormally upregulated in bladder cancer cell lines and tissues and associated with a poor prognosis in bladder cancer. Then, we showed that RNF26 promotes the progression of bladder cancer in vitro and in vivo. In addition, we demonstrated that aberrant overexpression of FOXM1 is responsible for the upregulation of RNF26 in bladder cancer cells through the MuvB complex. Moreover, we found that RNF26 interacts with p57 and decreases the stability of p57 to enhance the aggressiveness of bladder cancer cells (Fig. 7). Therefore, we uncovered a novel FOXM1/RNF26/p57 axis in bladder cancer, which could be a candidate target for bladder cancer therapy.

## Supplementary information

Supplementary information

## Data Availability

The datasets used and/or analyzed during the current study are available from the corresponding authors (Xin Jin, jinxinxy2@csu.edu.cn) on reasonable request.

## References

[1] Babjuk M, Burger M, Comperat EM, Gontero P, Mostafid AH, Palou J (2019). European Association of Urology guidelines on non-muscle-invasive bladder cancer (TaT1 and Carcinoma In Situ) - 2019 update. Eur Urol.

[2] Barani M, Hosseinikhah SM, Rahdar A, Farhoudi L, Arshad R, Cucchiarini M, et al. Nanotechnology in Bladder Cancer: Diagnosis and Treatment. Cancers. 2021;13:2214. 10.3390/cancers13092214 PMC8125468 34063088

[3] Zhang XG, Zhang T, Li CY, Zhang MH, Chen FM (2018). CD164 promotes tumor progression and predicts the poor prognosis of bladder cancer. Cancer Med.

[4] Witjes JA, Bruins HM, Cathomas R, Comperat EM, Cowan NC, Gakis G (2021). European Association of Urology Guidelines on muscle-invasive and metastatic bladder cancer: summary of the 2020 guidelines. Eur Urol.

[5] Swatek KN, Komander D (2016). Ubiquitin modifications. Cell Res.

[6] Faktor J, Pjechova M, Hernychova L, Vojtesek B (2019). Protein ubiquitination research in oncology. Klin Onkol.

[7] Berndsen CE, Wolberger C (2014). New insights into ubiquitin E3 ligase mechanism. Nat Struct Mol Biol.

[8] Deshaies RJ, Joazeiro CA (2009). RING domain E3 ubiquitin ligases. Annu Rev Biochem.

[9] Fenech EJ, Lari F, Charles PD, Fischer R, Laetitia-Thezenas M, Bagola K, et al. Interaction mapping of endoplasmic reticulum ubiquitin ligases identifies modulators of innate immune signalling. Elife. 2020;9::e57306. 10.7554/eLife.57306 PMC7332293 32614325

[10] Jongsma ML, Berlin I, Wijdeven RH, Janssen L, Janssen GM, Garstka MA (2016). An ER-associated pathway defines endosomal architecture for controlled cargo transport. Cell.

[11] Katoh M (2001). Molecular cloning and characterization of RNF26 on human chromosome 11q23 region, encoding a novel RING finger protein with leucine zipper. Biochem Biophys Res Commun.

[12] Jin X, Ding D, Yan Y, Li H, Wang B, Ma L (2019). Phosphorylated RB promotes cancer immunity by inhibiting NF-kappaB activation and PD-L1 expression. Mol Cell.

[13] Roh YG, Mun JY, Kim SK, Park W, Jeong MS, Kim TN, et al. Fanconi anemia pathway activation by FOXM1 is critical to bladder cancer recurrence and anticancer drug resistance. Cancers. 2020;12:1417. 10.3390/cancers12061417 PMC7352315 32486251

[14] Rubio C, Martinez-Fernandez M, Segovia C, Lodewijk I, Suarez-Cabrera C, Segrelles C (2019). CDK4/6 inhibitor as a novel therapeutic approach for advanced bladder cancer independently of RB1 status. Clin Cancer Res.

[15] Littler DR, Alvarez-Fernandez M, Stein A, Hibbert RG, Heidebrecht T, Aloy P (2010). Structure of the FoxM1 DNA-recognition domain bound to a promoter sequence. Nucleic Acids Res.

[16] Chen X, Muller GA, Quaas M, Fischer M, Han N, Stutchbury B (2013). The forkhead transcription factor FOXM1 controls cell cycle-dependent gene expression through an atypical chromatin binding mechanism. Mol Cell Biol.

[17] Engeland K (2018). Cell cycle arrest through indirect transcriptional repression by p53: I have a DREAM. Cell Death Differ.

[18] Litovchick L, Sadasivam S, Florens L, Zhu X, Swanson SK, Velmurugan S (2007). Evolutionarily conserved multisubunit RBL2/p130 and E2F4 protein complex represses human cell cycle-dependent genes in quiescence. Mol Cell.

[19] Marceau AH, Felthousen JG, Goetsch PD, Iness AN, Lee HW, Tripathi SM (2016). Structural basis for LIN54 recognition of CHR elements in cell cycle-regulated promoters. Nat Commun.

[20] Joaquin M, Gubern A, Gonzalez-Nunez D, Josue Ruiz E, Ferreiro I, de Nadal E (2012). The p57 CDKi integrates stress signals into cell-cycle progression to promote cell survival upon stress. EMBO J.

[21] Borges KS, Arboleda VA, Vilain E (2015). Mutations in the PCNA-binding site of CDKN1C inhibit cell proliferation by impairing the entry into S phase. Cell Div.

[22] Qin Y, Zhou MT, Hu MM, Hu YH, Zhang J, Guo L (2014). RNF26 temporally regulates virus-triggered type I interferon induction by two distinct mechanisms. PLoS Pathog.

[23] Mitra AP, Cote RJ (2009). Molecular pathogenesis and diagnostics of bladder cancer. Annu Rev Pathol.

[24] Mitra AP, Datar RH, Cote RJ (2006). Molecular pathways in invasive bladder cancer: new insights into mechanisms, progression, and target identification. J Clin Oncol.

[25] Mitra AP, Hansel DE, Cote RJ (2012). Prognostic value of cell-cycle regulation biomarkers in bladder cancer. Semin Oncol.

[26] Borriello A, Caldarelli I, Bencivenga D, Criscuolo M, Cucciolla V, Tramontano A (2011). p57(Kip2) and cancer: time for a critical appraisal. Mol Cancer Res.

[27] Kavanagh E, Joseph B (2011). The hallmarks of CDKN1C (p57, KIP2) in cancer. Biochim Biophys Acta.

[28] Stampone E, Caldarelli I, Zullo A, Bencivenga D, Mancini FP, Della Ragione F, et al. Genetic and epigenetic control of CDKN1C expression: importance in cell commitment and differentiation, tissue homeostasis and human diseases. Int J Mol Sci. 2018;19:1055. 10.3390/ijms19041055 PMC5979523 29614816

[29] Naito M, Mori M, Inagawa M, Miyata K, Hashimoto N, Tanaka S (2016). Dnmt3a regulates proliferation of muscle satellite cells via p57Kip2. PLoS Genet.

[30] Algar EM, Muscat A, Dagar V, Rickert C, Chow CW, Biegel JA (2009). Imprinted CDKN1C is a tumor suppressor in rhabdoid tumor and activated by restoration of SMARCB1 and histone deacetylase inhibitors. PLoS ONE.

[31] Cucciolla V, Borriello A, Criscuolo M, Sinisi AA, Bencivenga D, Tramontano A (2008). Histone deacetylase inhibitors upregulate p57Kip2 level by enhancing its expression through Sp1 transcription factor. Carcinogenesis.

[32] Heinen A, Tzekova N, Graffmann N, Torres KJ, Uhrberg M, Hartung HP (2012). Histone methyltransferase enhancer of zeste homolog 2 regulates Schwann cell differentiation. Glia.

[33] Chen C, Matesic LE (2007). The Nedd4-like family of E3 ubiquitin ligases and cancer. Cancer Metastasis Rev.

[34] Nicholson J, Jevons SJ, Groselj B, Ellermann S, Konietzny R, Kerr M (2017). E3 ligase cIAP2 mediates downregulation of MRE11 and radiosensitization in response to HDAC inhibition in bladder cancer. Cancer Res.

[35] Zhu J, Li Y, Chen C, Ma J, Sun W, Tian Z (2017). NF-kappaB p65 overexpression promotes bladder cancer cell migration via FBW7-mediated degradation of RhoGDIalpha protein. Neoplasia.

[36] Xu H, Ju L, Xiong Y, Yu M, Zhou F, Qian K (2021). E3 ubiquitin ligase RNF126 affects bladder cancer progression through regulation of PTEN stability. Cell Death Dis.

[37] Wenmaekers S, Viergever BJ, Kumar G, Kranenburg O, Black PC, Daugaard M, et al. A potential role for HUWE1 in modulating cisplatin sensitivity. Cells. 2021;10:1262. 10.3390/cells10051262 PMC8160634 34065298

