# Supplementary material for: The FOXM1/RNF26/p57 axis regulates the cell cycle to promote the aggressiveness of bladder cancer
Source: Cell Death Dis. 2021 Oct 14;12(10):944. doi: 10.1038/s41419-021-04260-z (PMC8516991; doi:10.1038/s41419-021-04260-z)
Supplement: Supplementary file 1 — Supplementary information [file 41419_2021_4260_MOESM1_ESM.docx]

**The FOXM1/RNF26/p57 axis regulates the cell cycle to promote the aggressiveness of bladder cancer**

Lu Yi, Haohui Wang, Wei Li, Kun Ye, Wei Xiong, Haixin Yu, Xin Jin

**
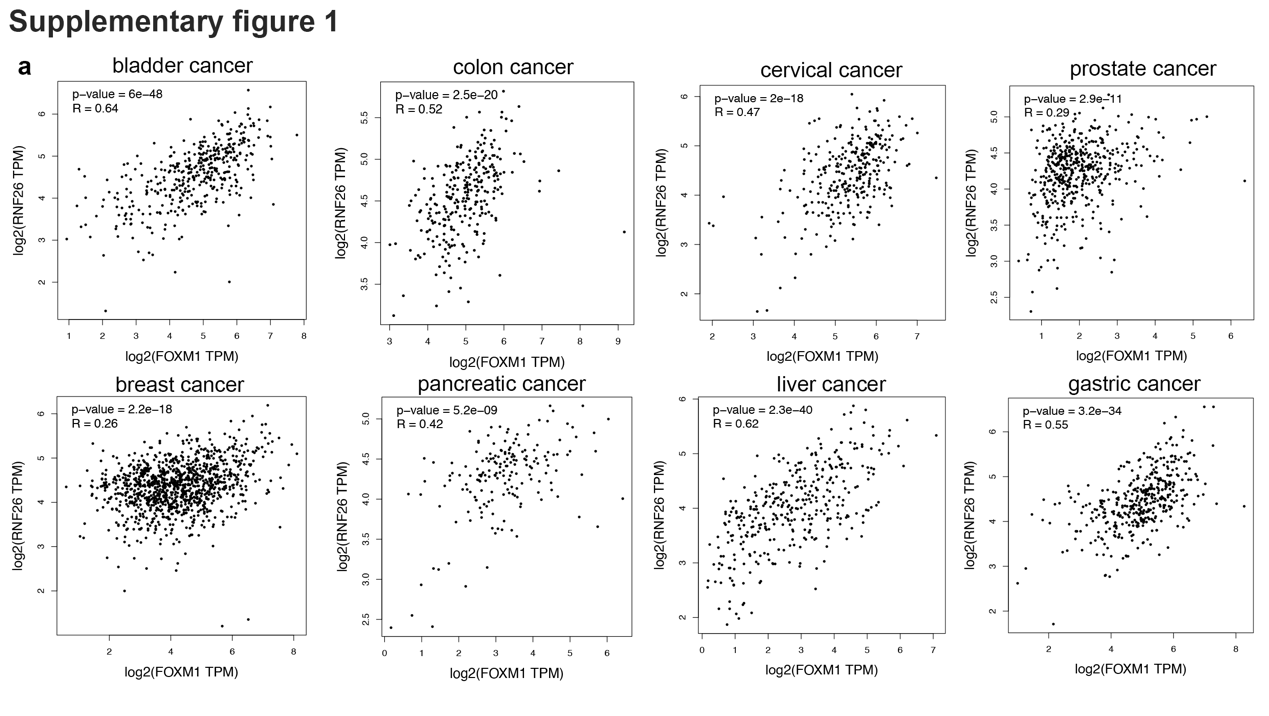
**

**Supplementary figure 1**

**a**, the correlation between RNF26 and FOXM1 were analyzed in multiple types of cancer by using the GEPIA web tool. Spearman correlation was preformed and r and P values were shown in figure.

**Supplementary Material and Methods**

**Public datasets for data mining and bioinformatics analysis**

TCGA-BLCA：Transcriptome data and clinical information of LUAD patients were obtained from the GDC data portal (<https://portal.gdc.cancer.gov/>). Data from 405 BLCA patients and 60488 genes were acquired. Of these patients, 19 samples had matched normal tissues.

**Survival analysis**

BLCA patients were divided into two groups according to the median expression level of the key gene. The differences in RFS and OS between the high and low expression groups were evaluated by the Kaplan–Meier method, followed by a log-rank test.

**GSEA for the key gene**

GSEA: BLCA patients were first divided into two groups according to the median expression level of the key gene. Then, differential expression analysis was applied between the high and low expression groups. Input genes for GSEA were sorted by their logFC values. Signaling pathways activated or suppressed by the key gene were decided by the normalized enrichment score (NES) value derived from GSEA.

ssGSEA: ssGSEA was used to calculate separate enrichment scores for each pairing of a BLCA sample and KEGG gene set. The ssGSEA score was further rescaled by min-max normalization method. Correlation analysis was performed between expression values of key gene and NES of signaling pathways.

**Upstream targets of the key gene**

Upstream targets of the key gene were determined by ChIP-seq and correlation analysis. Binding site in the targeted gene promoter of the key protein or methylation were obtained from ChIP-Atlas database (<https://chip-atlas.org/>). Correlation analysis between gene/protein and targeted genes was applied to further validate the ChIP-seq results.

**Predicted substrates for E3 ligase**

UbiBrowser ([http://ubibrowser.ncpsb.org.cn/](https://chip-atlas.org/)) was used to query potential substrates of a given E3 Ligase.

**Statistical analysis and visualization**

Microsoft R Open v4.0.2 was used for data mining, bioinformatics analysis and visualization in transcriptomics data. IGV v2.9.0 was used for analysis and visualization of ChIP-seq data.

**Plasmids transfection**

Flag-RNF26 was constructed by cloning the cDNA of RNF26 into the OmicLinkTM Expression Clone (CMV Promoter) (GeneCopoeia, EX-V0006-M14, USA). FOXM1 plasmids were purchased from WZ bioscience Inc. (Shangdong, China). The KOD-Plus-Mutagenesis Kit (#SMK-101, TOYOBO Life Science, Japan) was used to generate mutants of Flag-RNF26.

**Luciferase Reporter Assay**

GV592-RNF26 promoter reporter plasmids WT (- 450bp) were constructed into GV592 backbone (MCS-SV40-firefly_luciferase-PolyA-Tk-Renila_Luciferase-PolyA) by GENECHEM (Shanghai, China). The mutant GV592-RNF26 promoter reporter plasmids were generated by using the KOD-Plus- Mutagenesis Kit (Toyobo, Japan). The GV592-RNF26 promoter reporter plasmid was transfected into cells by using the Lipofectamine 2000 (Thermo Fisher Scientific, USA). Forty eight hours post transfection, cells harvested and tested with the Dual Luciferase Reporter Gene Assay Kit (Beyotime, RG027). All experiments were performed in three times.

**Quantitative real-time PCR and chromatin immunoprecipitation (ChIP)-qPCR**

TRIzol reagent (Thermo Fisher Scientific, USA) was used to extract the total RNA from cells. A reverse transcription kit (#RR037A PrimeScript™ RT reagent Kit, Takara Bio Inc. Shigo, Japan) and PCR kit (#RR430A, TB Green™ Fast qPCR Mix, Takara Bio Inc. Shigo, Japan) were used to perform RT-qPCR according to the manufacturer’s instructions reported previously ^1^. All measured values were normalized to that of GAPDH, and the 2^-ΔΔCt^ method was used to quantify fold changes. A chromatin extraction kit (Abcam, ab117152, USA) and the ChIP Kit Magnetic - One Step (Abcam, ab156907, USA) were used to perform ChIP following the manufacturer’s instructions reported previously ^1^. FOXM1 (Proteintech, #13147-1-AP, 1:100 dilution) was used for the ChIP assay. The primer sequences for RT-PCR and ChIP-qPCR are provided in Tables S2 and S3.

**Tissue microarray and immunohistochemistry (IHC)**

Tissue microarray slides (#U100Bl01) were purchased from Bioaitech, China. The tissue microarray specimens were immunostained with RNF26 (Proteintech, #16802-1-AP, 1:1000 dilution) and p57 (Proteintech, #23317-1-AP, 1:3000 dilution) antibody. The method of scoring the staining intensity was described previously ^2^.

**Cell proliferation assay**

For the Cell Counting Kit-8 (CCK-8) assay, 3000 cells were plated in 96-well plates cultured with 200 μl DMEM (Gibco, USA) containing 10% FBS for 5 days. Twenty microliters of CCK-8 reagent (#C0037, Beyotime) was added to each well one hour before the end of the incubation period following the manufacturer’s instructions. The optical density of each well at an absorbance of 450 nm was measured with a microplate reader.

**Reference**

1. Zhao J, Meng Z, Xie C, Yang C, Liu Z, Wu S*, et al.* B7-H3 is regulated by BRD4 and promotes TLR4 expression in pancreatic ductal adenocarcinoma. *Int J Biochem Cell Biol* 2019, **108:** 84-91.

2. Jin X, Ding D, Yan Y, Li H, Wang B, Ma L*, et al.* Phosphorylated RB Promotes Cancer Immunity by Inhibiting NF-kappaB Activation and PD-L1 Expression. *Mol Cell* 2019, **73**(1)**:** 22-35 e26.

**Table S1. The shRNA sequences.**

| ShRNF26 #1 | 5'-CCGGCACCGCGGAGTCTTGCTTTCATTGCTTCAAGAGAGCAATGAAAGCAAGACTCCGCTTTTTG-3' |
| --- | --- |
| ShRNF26 #2 | 5'-CCGGCACCGCCGTGGTCCGGTTCACATGTTTCAAGAGAACATGTGAACCGGACCACGGCTTTTTG-3' |
| ShRNF26 #3 | 5'-CCGGCACCGGAGCCTCCAGGTGGGCAAGATTCAAGAGATCTTGCCCACCTGGAGGCTCCTTTTTT-3' |
| ShFOXM1 #1 | 5'-CCGGCACCGCTCCGCCGGCGCCAATTTCATTCAAGAGATGAAATTGGCGCCGGCGGAGCTTTTTG-3' |
| ShFOXM1 #2 | 5'-CCGGCACCGCCGGCGCCAATTTCAAACAGTTCAAGAGACTGTTTGAAATTGGCGCCGGCTTTTTT-3' |
| Shp57 #1 | 5'-CCGGCACCGGCCTGAGCGAGCGAGCTAGCTTCAAGAGAGCTAGCTCGCTCGCTCAGGCCTTTTT-3' |
| Shp57 #2 | 5'-CCGGCACCGCGAGCGAGCTAGCCAGCAGGTTCAAGAGACCTGCTGGCTAGCTCGCTCGCTTTTT-3' |

**Table S2. The primer sequences for RT-qPCR.**

| Gene（Human） | Forward primer (5′ - 3′) | Reverse primer (5′ - 3′) |
| --- | --- | --- |
| GAPDH | ATGACAATGAATACGGCTACAGCA | GCAGCGAACTTTATTGATGGTATT |
| RNF26 | TCGGCACTCAGAACCTCTTT | GCCAGTGAGATTGACAAGCA |
| FOXM1 | CTGTTCAAAATGCCCCAAGT | TGCTGTGATGATGCTGTGAA |
| CDKN1C | CACGATGGAGCGTCTTGTC | CCTGCTGGAAGTCGTAATCC |

**Table S3. The primer sequences for ChIP-qPCR.**

| Gene | Forward primer (5′ - 3′) | Reverse primer (5′ - 3′) |
| --- | --- | --- |
| RNF26 primer1 | ATTGAGGGGAGTCCAAGACC | TCACTCATTGGTCCGTTTGA |
